# Perfect Absorption and Refractive-Index Sensing by Metasurfaces Composed of Cross-Shaped Hole Arrays in Metal Substrate

**DOI:** 10.3390/nano11010063

**Published:** 2020-12-29

**Authors:** Zhendong Yan, Chaojun Tang, Guohua Wu, Yumei Tang, Ping Gu, Jing Chen, Zhengqi Liu, Zhong Huang

**Affiliations:** 1 College of Science, Nanjing Forestry University, Nanjing 210037, China; zdyan@njfu.edu.cn; 2 Center for Optics and Optoelectronics Research, Collaborative Innovation Center for Information Technology in Biological and Medical Physics, College of Science, Zhejiang University of Technology, Hangzhou 310023, China; 3 College of Electronic and Optical Engineering, Nanjing University of Posts and Telecommunications, Nanjing 210023, China; 1219023306@njupt.edu.cn (G.W.); 1219023305@njupt.edu.cn (Y.T.); guping@njupt.edu.cn (P.G.); 4 College of Physics Communication and Electronics, Jiangxi Normal University, Nanchang 330022, China; 5 College of Physics and Electronic Engineering, Jiangsu Second Normal University, Nanjing 210013, China; huangzhong89@126.com

**Keywords:** perfect absorption, refractive-index sensing, metasurfaces

## Abstract

Achieving perfect electromagnetic wave absorption with a sub-nanometer bandwidth is challenging, which, however, is desired for high-performance refractive-index sensing. In this work, we theoretically study metasurfaces for sensing applications based on an ultra-narrow band perfect absorption in the infrared region, whose full width at half maximum (*FWHM*) is only 1.74 nm. The studied metasurfaces are composed of a periodic array of cross-shaped holes in a silver substrate. The ultra-narrow band perfect absorption is related to a hybrid mode, whose physical mechanism is revealed by using a coupling model of two oscillators. The hybrid mode results from the strong coupling between the magnetic resonances in individual cross-shaped holes and the surface plasmon polaritons on the top surface of the silver substrate. Two conventional parameters, sensitivity (*S*) and figure of merit (*FOM*), are used to estimate the sensing performance, which are 1317 nm/RIU and 756, respectively. Such high-performance parameters suggest great potential for the application of label-free biosensing.

## 1. Introduction

Recently, there is increasing interesting in studying the perfect absorption of electromagnetic waves for refractive-index sensing by employing metasurfaces [1,2,3,4,5,6,7,8,9,10,11,12,13,14,15,16,17,18,19,20,21,22,23,24,25,26,27,28,29,30,31,32], metallic nanostructures [33,34,35,36,37,38,39,40,41,42,43,44,45,46,47,48,49,50,51,52,53,54,55,56,57,58,59,60,61], and graphene nanostructures [62,63,64,65,66,67,68,69,70,71], in violet [62], visible [1,2,3,4,5,33,34,35,36,37,38,39,40,41,42,43,63,64], infrared [6,7,8,9,10,11,12,13,14,15,16,17,44,45,46,47,48,49,50,51,52,53,54,55,56,57,65], terahertz (THz) [18,19,20,21,22,23,24,58,59,60,61,66,67,68,69,70,71], and gigahertz (GHz) [25,26] frequency regions. In artificial metasurfaces, magnetic resonance is able to induce a substantial magnetic dipole to interact with the magnetic field of incident electromagnetic waves, and thus produce an effective permeability [72]. When the impedance of artificial metasurfaces is matched with that of vacuum, the incident electromagnetic waves will be nearly completely absorbed at a certain frequency range [72]. At present, most reported metasurfaces for perfect absorption and sensing are usually composed of a periodic array of metal nanoparticles with various shapes on the top surface of a dielectric film that is deposited on a metal substrate [1,2,3,4,5,6,7,8,9,10,11,12,13,14,15,16,17,18,19,20,21,22,23,24,25,26]. For example, Cong et al. demonstrated experimentally the metasurface electromagnetic wave perfect absorber, which consists of a square array of cross-shaped aluminum nanoparticles on a polyimide spacer supported on a very thick aluminum layer [19]. The remarkable enhancement of electromagnetic fields at the magnetic dipolar resonance enables a very strong interaction with the analyte for ultrasensitive sensing scheme in THz frequencies.

For metal nanostructures, the well-known surface plasmon resonances or the other resonance modes are able to result into the huge electromagnetic field enhancement, which can be also utilized for the perfect absorption and refractive-index sensing [33,34,35,36,37,38,39,40,41,42,43,44,45,46,47,48,49,50,51,52,53,54,55,56,57,58,59,60,61]. For example, Shi et al. theoretically studied a multi-band perfect absorber for sensing application in visible and near-infrared regions, which is based on the excitations of localized surface plasmons, delocalized surface plasmon polaritons, and lattice plasmon resonances, in the periodic arrays of gold nanodisks with a prismatic hole standing on silica/gold bilayer films [36]. Bhattarai et al. experimentally investigated the refractive-index sensing in near-infrared region, when the perfect absorption is obtained by Fabry–Perot cavity mode in the periodic arrays of gold elliptical nanodisks lifted by dielectric pillars on gold substrate [46].

From mid-infrared to THz frequencies, nanostructured graphene is capable of supporting surface plasmon resonances [73], because graphene has an effective permittivity of standard Drude model. The plasmon resonances of graphene nanostructures have narrower bandwidths due to their low damping rates and, thus, result into stronger electromagnetic field confinement, as compared with those in metal nanostructures [74]. More importantly, the positions of the plasmon resonances graphene can be tuned via bias voltage [75]. These properties make nanostructured graphene a good candidate for perfect absorption and sensing application [62,63,64,65,66,67,68,69,70,71]. For example, Yi et al. propose a graphene-based plasmonic sensor with dual-band perfect absorption in infrared region, for the periodically arranged graphene nanorings to be placed on the silica film deposited on the gold substrate [69]. The position of dual-band perfect absorption can be shifted by applying an external voltage on graphene to manipulate the Femi energy, which is favorable for more flexible sensing applications.

The change of the refractive index of the environment medium will lead to the obvious shift of the positions of surface plasmon resonances or magnetic resonances, and the corresponding spectral intensity such as transmission and reflection will also undergo a change. This is the fundamental physical mechanism of the refractive-index sensing. The major advantage of perfect absorption for sensing is that a very small change of the refractive index is able to be detected [10], because the spectral position and intensity will exhibit a dramatic change if the utilized resonance mode has a narrow bandwidth and a strong enhancement of electromagnetic fields. However, as pointed out recently by Yong et al. [56], simultaneous achieving the ultra-narrow band perfect absorption and the very large enhancement of electromagnetic fields is challenging, owing to the fast radiative damping of the resonance mode and the intrinsic Ohmic loss in metals.

In this work, we numerically demonstrate an ultra-narrow band perfect absorption with a significant electromagnetic field enhancement in near-infrared region, whose *FWHM* is only 1.74 nm. The ultra-narrow band perfect absorption results from the hybrid mode of magnetic resonance and surface plasmon polaritons in metasurfaces composed of a periodic array of cross-shaped holes in a silver substrate. The resonant position of the hybrid mode can be predicted well by using a coupling model of two oscillators. Numerical results show that two conventional performance parameters for sensing, *S* and *FOM*, have very high values of 1317 nm/RIU and 756, respectively, which suggests great potential for the application of label-free biosensing.

## 2. Methods

**Figure 1:**
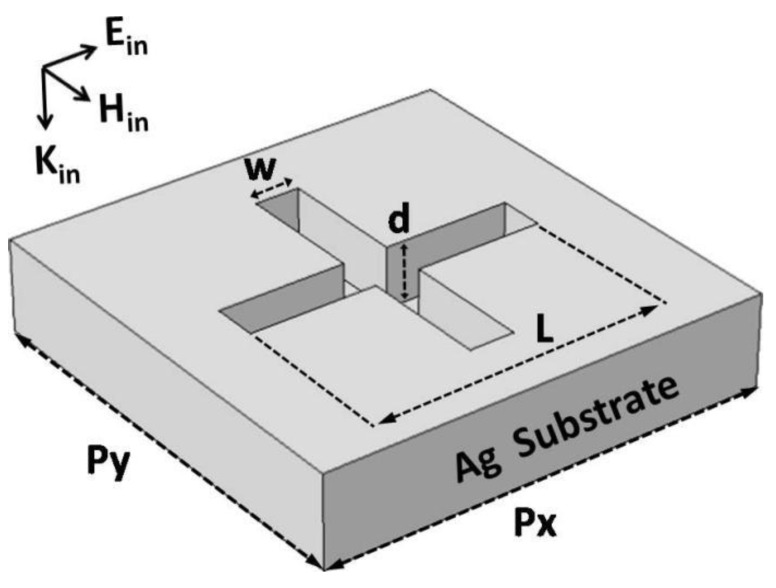
Schematic of the building block of metasurfaces for perfect absorption and refractive-index sensing.

In Figure 1, we schematically show the unit cell of our studied metasurfaces, which are composed of a periodic array of cross-shaped holes drilled in a silver substrate. The length, width, and depth of the cross-shaped holes are *l*, *w*, and *d*, respectively, as indicated by the arrowed lines. The array periods along the *x*-axis direction and the *y*-axis direction are *p_x_* and *p_y_*, respectively. The light (electromagnetic) wave is supposed to be normally incident from top to bottom. The electric field (***E****_in_*), the magnetic field (***H****_in_*), and the wave vector (***k***) of incident light are along the *x*-axis direction, the *y*-axis direction, and the negative *z*-axis direction, respectively, as indicated by three arrows in the left upper corner. In this work, we will use the commercial software package “EastFDTD” [https://www.eastfdtd.com] to calculate the absorption spectra and the electromagnetic field distributions. In numerical simulations, the silver film has a finite thickness of 500 nm. The calculated absorption spectra are completely the same, provided that the silver film is enough thick and thus the incident light could not pass through it. Even if the silver film with sufficient thickness is supposed to be deposited on a dielectric substrate, the calculated absorption spectra are still the same, because the incident light is not able penetrate the silver film to feel the existent of the dielectric substrate. Periodic boundary condition is applied to the *x* and *y*-axis directions, and perfectly matched layer is set along the *z*-axis direction. For numerical convergence, the mesh size Δ*s* is taken to be 5 and 50 nm in the cross-shaped hole and the other region, respectively. The time step is Δ*t* = Δ*s*/2*c*, where *c* is light speed in vacuum. More information about the commercial software package can be found at the website (https://www.eastfdtd.com). In our calculations, experimental data are used for the wavelength-dependent real and imaginary parts of complex refractive index of silver substrate [76]. The proposed metasurfaces can be experimentally fabricated by focused ion beam (FIB) lithography. The commonly used FIB setup (Strata FIB 201, FEI Company, Hillsboro, OR, USA) is able to mill the proposed array of cross-shaped holes on a silver film on a quartz substrate. The thick silver film is firstly deposited on a quartz substrate by electron beam evaporation before the process of the FIB lithography. The linear optical response of reflection (R) of our proposed metasurfaces is measured experimentally by using a commercial Fourier-transform infrared spectrometer (FTIR, Nicolet 6700) equipped with a polarizer. Then, the absorption (A) obtained in experiment is equal to 1—R.

## 3. Results and Discussion

**Figure 2:**
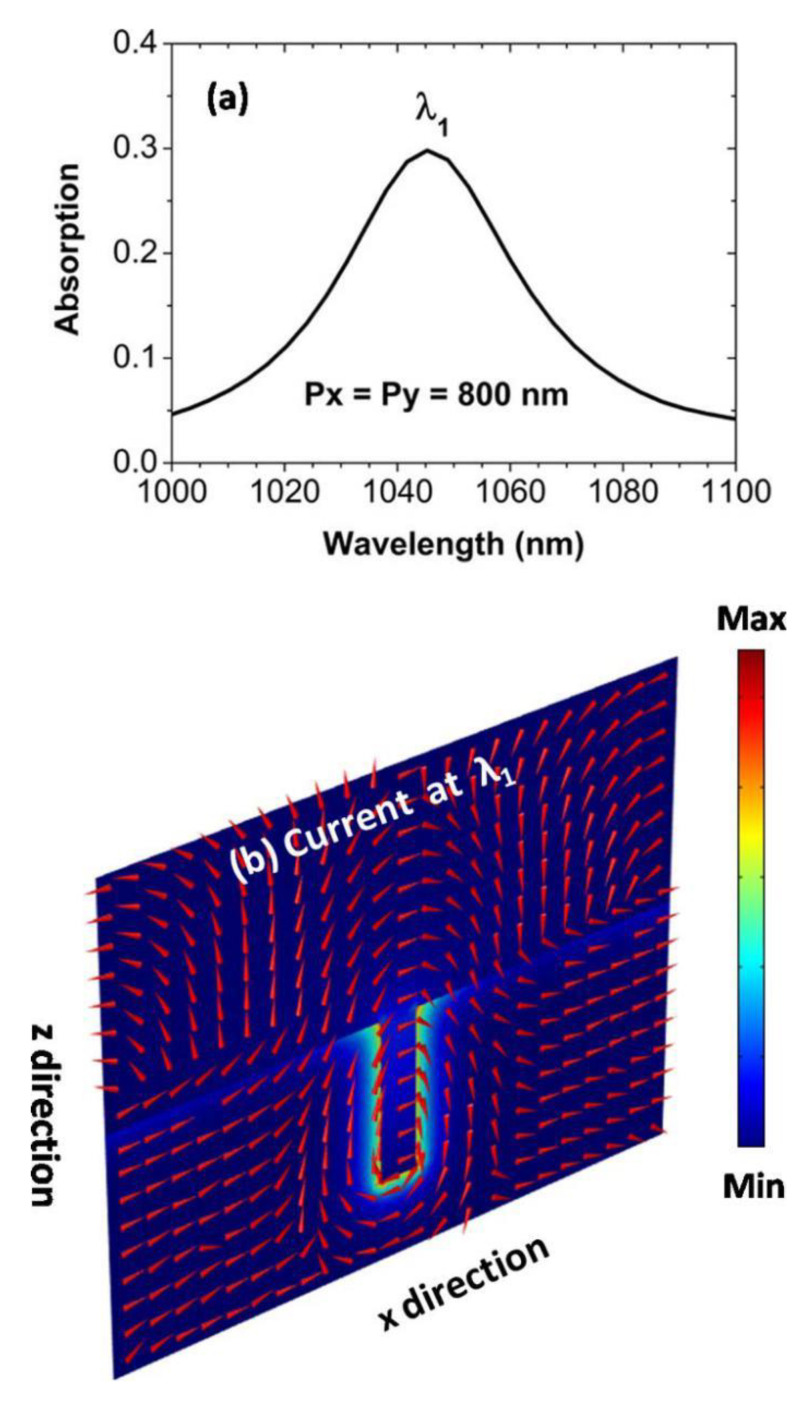
(**a**) Numerically calculated absorption spectra at normal incidence, with geometrical parameters of *p_x_* = *p_y_* = 800 nm, *L* = 500 nm, *d* = 200 nm, and *w* = 50 nm. (**b**) Current distributions at the wavelength of *λ*_1_ on the *xz* plane along the white dotted line in Figure 3a. The colors represent the current strength, and the red arrows give the current directions.

**Figure 3:**
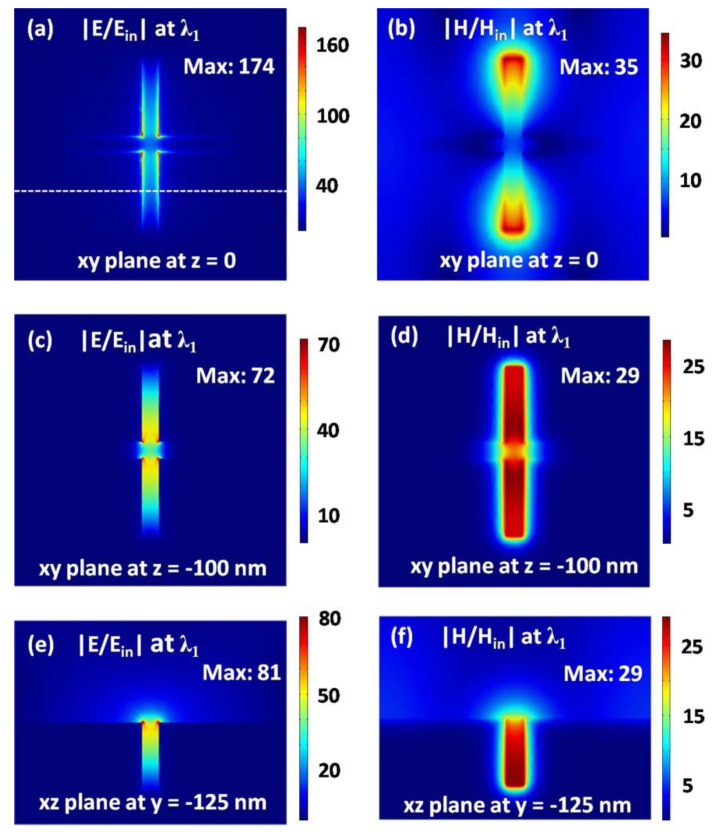
Electric (**a**,**c**,**e**) and magnetic (**b**,**d**,**f**) field strength at the resonance wavelength of *λ*_1_. The *xy* plane with *z* = 0 (**a**,**b**) is just on the top surface of the metal substrate. The *xy* plane with *z* = −100 nm (**c**,**d**) is at the geometrical center of the cross-shaped hole. The *xz* plane with *y* = −125 nm (**e**,**f**) is along the white dotted line in (**a**).

In Figure 2a, we show the numerically calculated absorption spectra at normal incidence, with the array periods *p_x_* = *p_y_* = 800 nm and the geometrical size of the cross-shaped holes *L* = 500 nm, *d* = 200 nm, and *w* = 50 nm. An absorption peak is clearly seen, which is centered at the wavelength of *λ*_1_ = 1045 nm. To reveal the physics of the absorption peak, in Figure 2b we show the current distributions at the wavelength of *λ*_1_ on the *xz* plane with *y* = −125 nm. Obviously, the currents near the cross-shaped hole are relatively stronger, and create a loop on the *xz* plane, as indicated by the red arrows in Figure 2b. The current loop will produce a substantial magnetic moment, which is able to response to the magnetic field of incident light, and thus form a magnetic resonance [77]. The magnetic resonance is the physical origin of the absorption peak centered at the wavelength of *λ*_1_. The forming mechanism of the magnetic resonance is very similar to that in the U-shape metallic split-ring-resonators (SRRs). In order to better understand the magnetic resonance, in Figure 3 we have plotted electric and magnetic field distributions at the wavelength of *λ*_1_ on three planes. In Figure 3a,b, the *xy* plane with *z* = 0 is just on the top surface of the silver substrate. In Figure 3c,d, the *xy* plane with *z* = −100 nm is at the geometrical center of the cross-shaped hole. In Figure 3e,f, the *xz* plane with *y* = −125 nm is along the white dotted line in Figure 3a.

**Figure 4:**
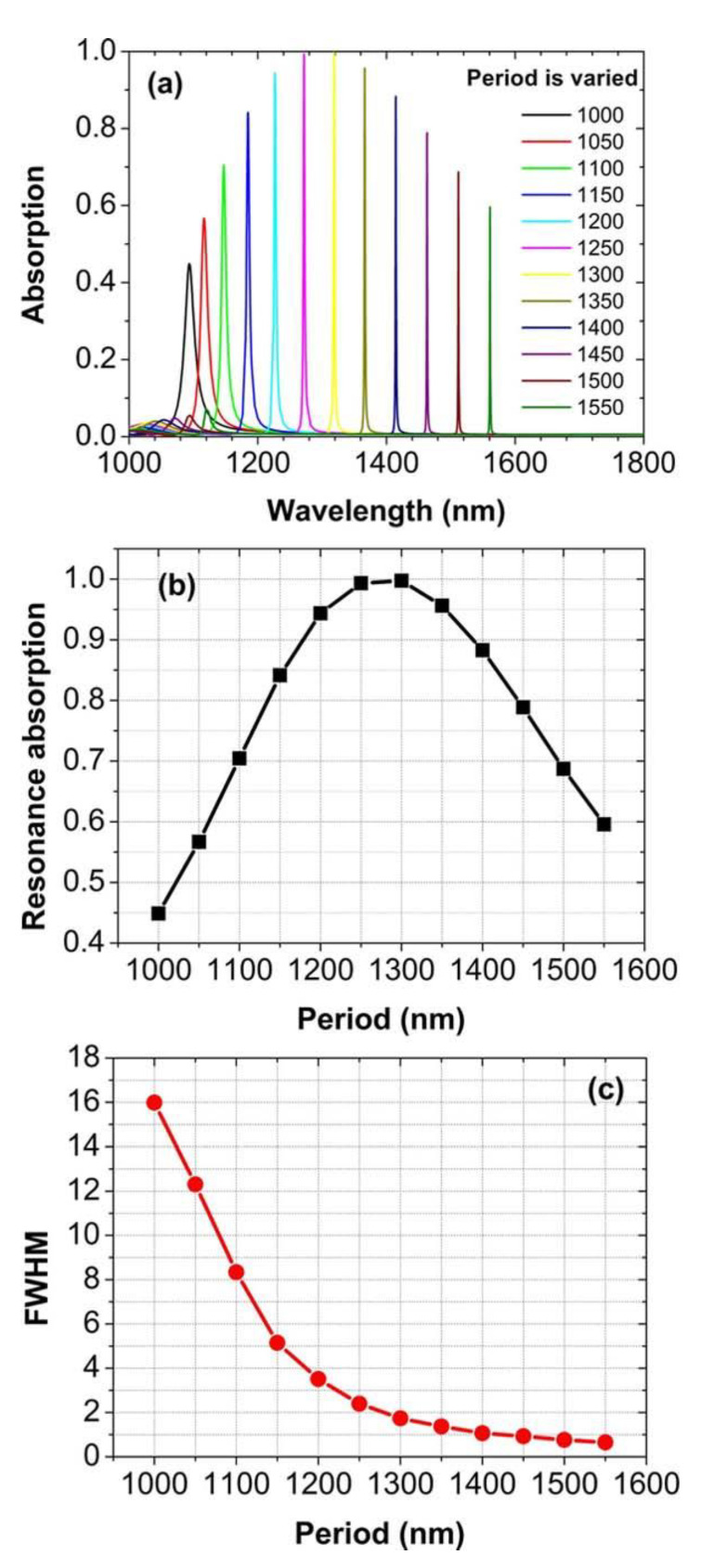
(**a**) Normal-incidence absorption spectra for the period (*p_x_* = *p_y_*) to be varied from 1000 to 1550 nm in steps of 50 nm. (**b**,**c**) Maximum value and *FWHM* of absorption peak for different periods, respectively.

In Figure 4a, we present a series of absorption spectra at normal incidence, for the array periods of both *p_x_* and *p_y_* to be increased from 1000 to 1550 nm in steps of 50 nm. For each period, there is a very sharp absorption peak, which red-shifts when the array periods are increased. Figure 4b shows the dependence of the maximum value at the absorption peak on the array period. At a proper period of 1300 nm, the nearly perfect absorption more than 99.7% is achieved. Away from the period of 1300 nm, the peak value will decrease quickly. The *FWHM* of the absorption peak for different periods is exhibited in Figure 4c. With the increasing period, the absorption peak becomes more and more narrow, whose *FWHM* can be reduced from about 16 nm to even 0.65 nm. For the perfect absorption peak, the *FWHM* is about 1.74 nm. Achieving such a perfect absorption peak with an extremely narrow bandwidth is desired for high-performance sensing applications.

Next, we will prove that the sharp absorption peak in Figure 4a is related to a hybrid mode, which results from the coupling between magnetic resonance localized within the cross-shaped hole and surface plasmon polaritons propagating on the top surface of the silver substrate. A coupling model of two oscillators can be used to calculate the energy of the hybrid mode [78], and the computing formula is:

(1) $$E_{+,-}=(E_{\mathrm{MR}}+E_{\mathrm{SPP}})/2\pm \sqrt{\Delta /2+{\left(E_{\mathrm{MR}}-E_{\mathrm{SPP}}\right)}^{2}/4}$$

**Figure 5:**
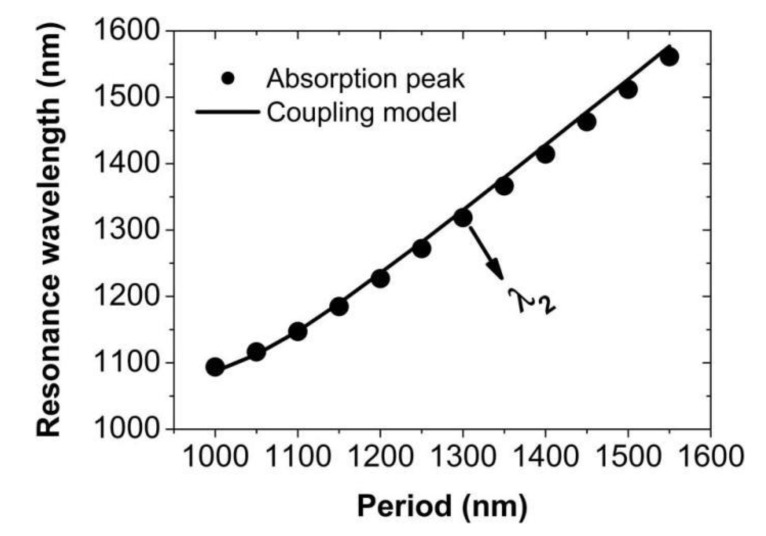
Comparison of the positions of absorption peaks with those predicted by the coupling model of two oscillators.

The energy of magnetic resonance, *E_MR_*, is 1.1866 eV, corresponding to the wavelength of *λ*_1_ = 1045 nm. The energy of surface plasmon polaritons, *E_SPP_*, can be analytically obtained for different periods [79]. In Figure 5, we have compared the positions of absorption peaks for different periods with the resonance wavelengths of the hybrid mode predicted by the above theoretical model with the coupling strength Δ = 0.008 eV. Obviously, they are in an excellent agreement.

**Figure 6:**
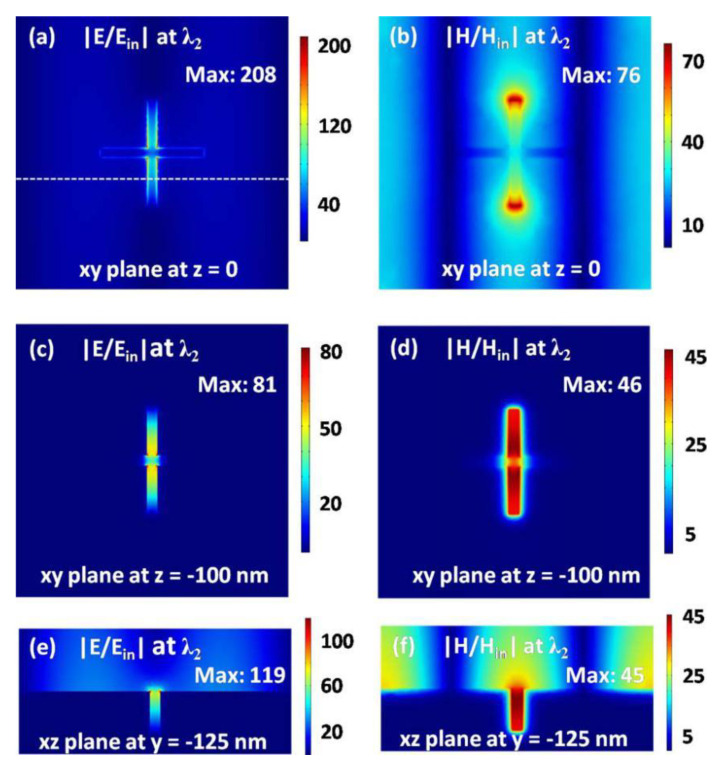
Electric (**a**,**c**,**e**) and magnetic (**b**,**d**,**f**) field strength at the resonance wavelength of *λ*_2_. The *xy* plane with *z* = 0 (**a**,**b**) is just on the top surface of the metal substrate. The *xy* plane with *z* = −100 nm (**c**,**d**) is at the geometrical center of the cross-shaped hole. The *xz* plane with *y* = −125 nm (**e**,**f**) is along the white dotted line in (**a**).

To further understand the property of the hybrid mode, in Figure 6 we plot the distributions of electric and magnetic fields at the resonance wavelength *λ*_2_ = 1318.7 nm of the perfect absorption with the array periods *p_x_* = *p_y_* = 1300 nm. The positions of three planes in Figure 6 are the same as those in Figure 3, but the array periods are different. The electromagnetic fields in Figure 6 get stronger on all three planes, as compared with those in Figure 3. Since the magnetic resonance is a localized mode associated with the cross-shaped hole, so the electromagnetic fields in Figure 3 are mainly distributed into the hole. However, for the hybrid mode we also observe the regular distribution pattern of electromagnetic field enhancement on the top surface of the silver substrate, as clearly seen in Figure 6a,b,e,f. The regular distribution pattern is very prominent in Figure 6e,f, which suggests the excitation of surface plasmon polaritons propagating on the silver surface [79]. The distribution property of electromagnetic fields further confirms that the sharp absorption peak origins from the hybrid mode, due to the coupling of magnetic resonance with surface plasmon polaritons. It is well known that surface plasmon polaritons have a slow radiation damping [79], and so the bandwidth of the hybrid mode is very narrow. The maximum amplitudes of electric and magnetic fields are 208 and 76 times of those of the incident light, respectively. Correspondingly, the maximum intensities of electric and magnetic fields, which are the square of amplitude, can be enhanced to 43,264 and 5776 times of those of the incident light. Such a high enhancement of electromagnetic fields is comparable with the values reported recently [56], which is helpful for sensing applications.

**Figure 7:**
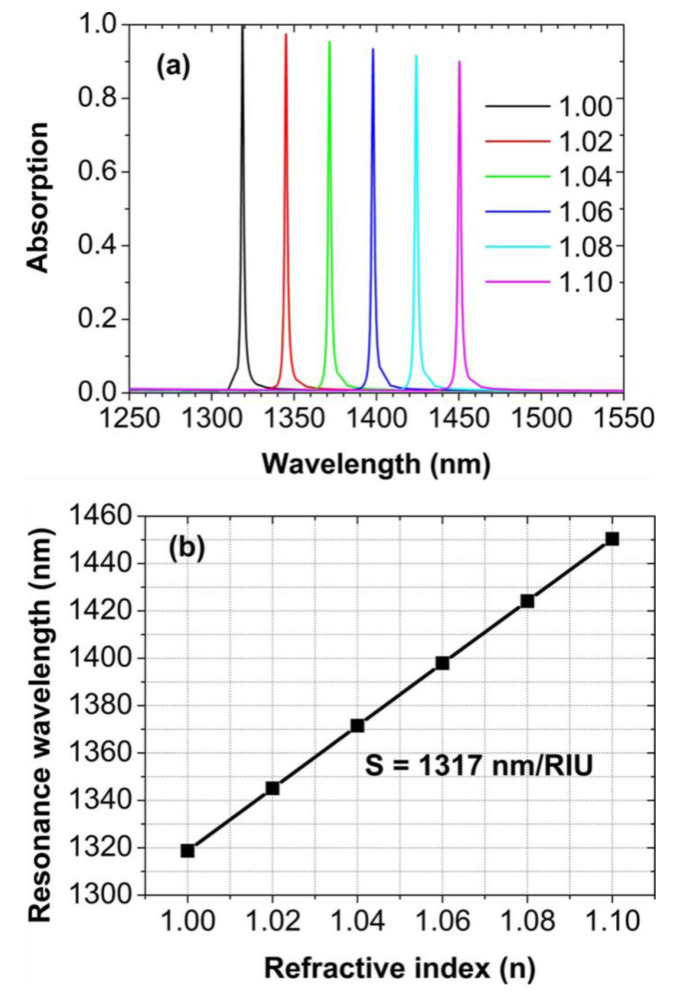
(**a**) Normal-incidence absorption spectra with the refractive index (*n*) of surrounding medium increased from 1.0 to 1.1 in steps of 0.02. (**b**) The position of absorption peak for different *n*.

To study the potential sensing applications of our studied nanostructures, in Figure 7a we show a series of absorption spectra at normal incidence with the array periods *p_x_* = *p_y_* = 1300 nm, for the refractive index of surrounding medium is varied from 1.0 to 1.1 in steps of 0.02. When the refractive index of surrounding medium is increased slightly, the absorption peak will shift obviously, which is very sensitive to the change of the refractive index. Figure 7b shows the dependence of the position of the absorption peak on the refractive index. Conventionally, two important parameters, sensitivity (*S*) and figure of merit (*FOM*), are widely used to estimate the sensing performance [80,81,82,83,84]. *S* and *FOM* can be defined as:

(2) S=Δλ/Δn,FOM=S/FWHM

where Δλ is the spectral shift of the absorption peak, Δ*n* is the change of the refractive index, and *FWHM* is the full width at half maximum of the perfect absorption peak. In our case, *S* = 1317 nm/RIU, which is the slope of the black line in Figure 7b. The *FWHM* of the perfect absorption peak is only 1.74 nm, so we achieve an ultra-high *FOM* of about 756. These two values of performance parameters are very high, which are far larger than those reported recently in many papers. In Table 1, we give *S* and *FOM* of sensors based on perfect absorption in the near-infrared region. The dephasing time of the hybrid mode, defined as 2 *ħ*/*FWHM* [85], is about 1.2 × 10^−25^ s.

## 4. Conclusions

In summary, we have studied theoretically metasurfaces for high-performance refractive-index sensing based on the ultra-narrowband perfect absorption of electromagnetic waves in infrared region. The studied metasurfaces are composed of a periodic array of cross-shaped holes in a silver substrate. The ultra-narrow band perfect absorption results from a hybrid mode, due to the coupling between the magnetic resonances localized within individual cross-shaped holes and the surface plasmon polaritons propagating on the top surface of the silver substrate. Numerical results show a high sensitivity (*S*) of 1317 nm/RIU and a large figure of merit (*FOM*) reaching to 756, which suggests great potential for the application of label-free biosensing.

## Figures and Tables

**Table 1:** Performance of sensors based on perfect absorption in the near-infrared region.

References	*λ* (nm)	*S* (nm/RIU)	FOM (RIU^−1^)
[12]	1711	1240.8	123.45
[13]	2103	1445	28.8
[14]	1649	260.4	2.91
[49]	1728	900	15
[50]	1159	959	16.54
[54]	1536	150	25
[55]	1541	1170	1054
[56]	918	885	110
[57]	907	991	124
This work	1318	1317	756

## Data Availability

All data have been illustrated in the manuscript and in the supplementary material.
